# Methylation-Sensitive Melt Curve Analysis of the *Reprimo* Gene Methylation in Gastric Cancer

**DOI:** 10.1371/journal.pone.0168635

**Published:** 2016-12-16

**Authors:** Hanze Wang, Yansong Zheng, Junzhong Lai, Qianping Luo, Huican Ke, Qi Chen

**Affiliations:** 1 Fujian Key Laboratory of Innate Immune Biology, Biomedical Research Center of South China, College of Life Science, Fujian Normal University Qishan Campus, Fujian, China; 2 The First Affiliated Hospital of Fujian Medical University, Fujian, China; University of Bristol, UNITED KINGDOM

## Abstract

*Reprimo (RPRM)* is a p53-induced tumor suppressor gene. Its aberrant DNA methylation is correlated with carcinogenesis and may be used as a surrogate marker for the early detection of gastric cancer. However, the detail information regarding its DNA methylation has not been revealed. Here, we investigated the *RPRM* gene methylation in gastric cancer tumor and plasma samples by methylation-sensitive melt curve analysis (MS-MCA) and bisulfite sequencing in depth. We developed a semi-quantitative method based on MS-MCA for detecting DNA methylation and unraveled the *RPRM* gene methylation pattern in gastric cancer. This study provides a solid foundation for the future application of detecting *RPRM* gene methylation in human plasma or serum samples to help diagnose gastric cancer or for prognosis evaluation.

## Introduction

Gastric cancer is the fourth most common cancer and the second leading cause of cancer-related death in the world [1]. Although early detection of gastric cancer increases the probability of a better prognosis, most cases of gastric cancer remain asymptomatic until the advanced stages when the best timing for intervention is gone. Therefore, the identification of applicable biomarkers for early detection of gastric cancer is an imperative challenge.

Both epigenetic and genetic abnormalities play important roles in carcinogenesis [2]. The epigenetic pathway is involved in the regulation of chromatin structures by various mechanisms, including DNA methylation, histone modifications, or noncoding RNA regulation [3]. In cancers, abnormal DNA methylation frequently occurs at the promoter regions of genes, especially tumor-suppressor ones, which affects nuclear organization, histone modifications, and linker histone binding [4].

Reprimo (TP53 dependent G2 arrest mediator candidate, RPRM) is a glycosylated cytoplasmic protein that was identified by differential display screening of genes derived from wild-type and p53/interferon regulatory factor-1-deficient mouse embryonal fibroblasts after X-irradiation, and thus is classified as a p53-inducible gene [5, 6]. Its gene, *RPRM*, is localized on chromosome 2q23 where the allelic imbalance is frequently observed in human cancers [7]. Overexpression of Reprimo induces the G2 arrest of the cell cycle accompanied by inhibition of both Cdc2 activities and cyclin B1 nuclear translocation [5]. The down-regulation of the *RPRM* transcript is associated with *RPRM* promoter methylation in some tumors and tumor cell lines [8, 9].

It has been reported that elevated circulating DNA levels in serum were found in various cancer entities which can help discriminate cancer patients from healthy individuals and patients with non-malignant diseases [10–12]. Furthermore, the characteristics of circulating DNA, such as derived from the genetic and epigenetic alterations, are preserved and are often in consonance with that found in the tumors [13, 14]. Thus, after successful retrieve from plasma or serum, the circulating tumor DNA can be used as a surrogate marker to analyze genetic or the epigenetic alterations present in the original tumor tissues.

Here, we investigated the *RPRM* gene methylation in gastric cancer tumor and plasma samples by methylation-sensitive melt curve analysis (MS-MCA) and bisulfite sequencing in depth, and analyzed the *RPRM* gene methylation pattern in gastric cancer and suggested a possible application of MS-MCA in analysis of clinical specimen’s pathological features.

## Materials and Methods

### Cell lines and clinical samples

Gastric cell lines used in the study, GES-1, AGS, BGC-823, SGC-7901 and MKN-45, were generously given by the Lab of Development Biology, College of Life Sciences, Fujian Normal University in 2014. Cells were passaged or cryopreserved following regular cell culture procedures after obtained.

All of the blood or tumor tissue samples were collected from the First Affiliated Hospital of Fujian Medical University. In total, 49 clinically-diagnosed gastric cancer patients, 29 non-tumor patients and 28 healthy adults participated in this project. Only 28 of the 49 clinically-diagnosed gastric cancer patients were procured with the complete sample sets (gastric cancer tissue, para-cancerous tissue and blood sample) due to the hospital’s administration. Among them, 14 patients contributed gastric cancer biopsy samples taken from gastroscopy and another 7 patients provided only blood (plasma) samples. As controls, blood samples were also extracted from 28 age-matched healthy adults without gastric abnormalities and 29 non-tumor patients who were free from malignant tumor invasion but diagnosed with gallstones, cholecystitis, and intestinal obstruction jaundice, etc. As for the 41 PBMC samples, we used the blood samples from 19 gastric cancer patients and 22 healthy adults for analysis. Collection of All of the blood or tumor tissue samples was approved by the medical ethics committee of the First Affiliated Hospital of Fujian Medical University (Fujian Medical University affiliated medical ethics review permit No. [2013]23) and following the signed consent agreements with patients. The surgeries of all of the cases were performed at their first time.

The gastric cancerous and the corresponding para-cancerous tissues were dissected during surgery, with the corresponding blood samples extracted as well. As controls, blood samples were also extracted from age-matched healthy adults or non-tumor patients who were free from malignant tumor invasion but diagnosed with gallstones, cholecystitis, and intestinal obstruction jaundice, *etc*.

Upon dissection, tissue samples were immersed into the sample protector solution (TAKARA BIOTECHNOLOGY, DALIAN, China) and stored at liquid nitrogen until use. For blood samples, the plasma and blood cells were stored at -80°C, separately, after centrifuge at 4,000 rpm for 5 min.

### DNA extraction and bisulfite conversion

DNA from tumor tissues was extracted using the universal genomic DNA extraction kit (TAKARA BIOTECHNOLOGY, DALIAN, China). Circulating DNA from plasma samples was isolated using ChargeSwitch® gDNA 1 ml the serum kit (Catalog no. CS11040, Life Technologies, USA) according to the manufacturer’s instruction. The DNA from blood cells, mostly peripheral blood mononuclear cells (PBMCs), was extracted by using the blood genome DNA extraction kit (TIANGEN Biotech, Beijing, China).

The above DNA samples were bisulfite converted and purified using EpiTect® plus bisulfite conversion kit (Qiagen, Germany) according to the manufacturer’s instruction. The purified DNA was then subjected to methylation-sensitive melt curve analysis (MS-MCA) or bisulfite sequencing as follows.

### Bisulfite sequencing

The CpG islands in the 5’-flanking region of *RPRM* gene (NCBI reference sequence: NC_000002.12) were predicted by MethPrimer [15] and we picked one pair of bisulfite sequencing primers (RPRM-F: 5’-GTTTTAGAAGAGTTTAGTTGTTG-3’; RPRM-R: 5’-CTACTATTAACCAAAAACAAAC-3’) which contains 30 C-G dinucleotides within the target sequence to study the *RPRM* gene methylation. The target reference sequences of human *RPRM* gene (Ref-seq), the corresponding bisulfite-converted sequences from fully-methylated (M-seq) or unmethylated (U-seq) genomic DNA were provided in S1 File.

For bisulfite sequencing, the PCR was conducted using the bisulfite primers and Ex Taq HS (TAKARA BIOTECHNOLOGY, DALIAN). The PCR products were purified using TaKaRa MiniBEST agarose gel DNA extraction kit (TAKARA BIOTECHNOLOGY, DALIAN) before integrated into the pGEM®-T Easy vector (Promega Biotech, Beijing). After transforming the *E*.*coli* DH5α competent cells, eight to ten clones were sequenced and then aligned with M-seq and U-seq for analysis of cytosine methylation using the BioEdit software [16].

### Methylation-Sensitive Melt Curve Analysis (MS-MCA) for unmethylated or methylated samples

**Fig 1:**
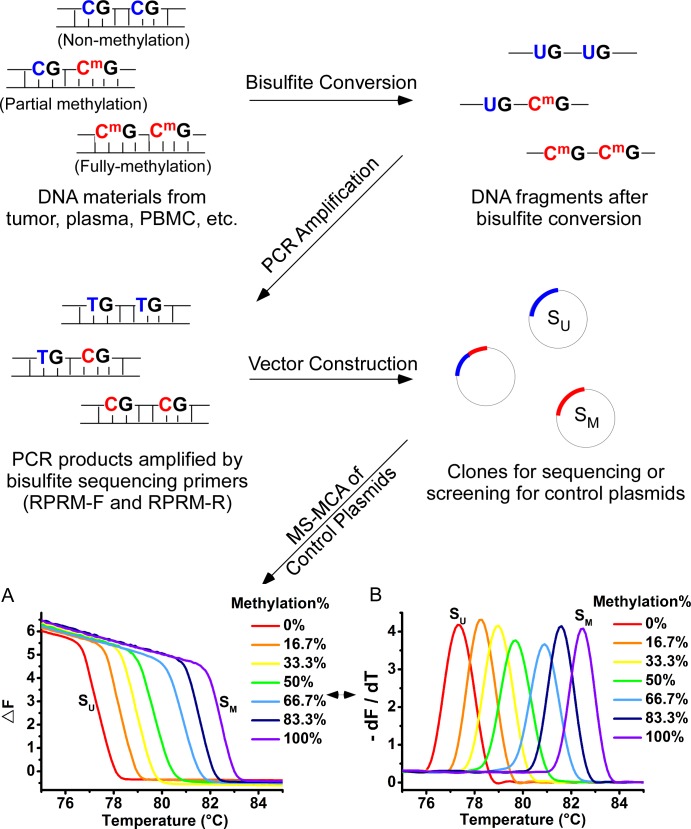
Flowchart for bisulfite sequencing of DNA samples and the screening of control plasmids. (A) Melt curves for each control plasmid simulating DNA with different methylation degrees, with X axis representing for temperature and Y axis for normalized fluorescence (△F). (B) The differential form of melt curves, with X axis representing for temperature and Y axis for a minus differentiation of normalized fluorescence to temperature.

For MS-MCA, PCR was performed using SYBR® Premix Ex Taq II (Tli RNaseH Plus) (TAKARA, DALIAN, China), and the conditions are as follows: the amplification stage: 95°C 30 s; [95°C 5 s, 56°C 15 s, 72°C 30 s] for 40 cycles; the melt curve stage: 95°C 15 s, 72 to 88°C with 0.1°C increment per cycle. Each plate was calibrated using two control plasmids obtained from bisulfite sequencing clones as mentioned in the above section, designated as S_U_ and S_M_ to pinpoint the unmethylated and fully-methylated melt curve peaks, respectively. The target sequences of these two plasmids were exactly the U-seq and M-seq sequences as mentioned above, respectively. And the construction schemes were shown in Fig 1.

Twenty cases of healthy adults’ PBMC samples were used to define the non-methylation region in the MS-MCA of *RPRM* gene. For all these curves, a dramatic decline of the fluorescence signals of the target PCR products began after 75.5°C, and ended before 79.5°C (S1A Fig). Therefore, the range from 75.5 to 79.5°C was set as a non-methylation region. Similarly, 20 independent runs of S_M_ were used to define the fully-methylation region, which ranges from 80.5 to 84.5°C (S1B Fig). Therefore, for those melt curves which completely contained within the range from Tm = 75.5°C to 79.5°C, the corresponding samples should be considered as unmethylated ones; for the melt curves covering the range beyond this scope but not exceeding 84.5°C, the samples were presumed to be methylated to a certain degree; and if the melt curves completely contained within the range from Tm = 80.5 to 84.5°C, the samples would be considered as fully methylated.

In addition, to test if MS-MCA was able to help determine the heterogeneity of a sample, we employed several control plasmids, S_0_ (*i*.*e*., S_U_), S_5_, S_10_, S_15_, S_20_, S_25_, and S_30_ (*i*.*e*., S_M_), mocking the samples methylated to the degrees of 0%, 16.7%, 33.3%, 50%, 66.7%, 83.3% and 100%, and mixed them in the different ways to simulate the heterogeneous methylation nature of a sample. The homogenous MS-MCA results of each of these control plasmids alone were indicated in Fig 1A and 1B. To simulate the heterogeneous results, we pooled 2 plasmids (S_0_ and S_30_), 3 plasmids (S_0_, S_15_ and S_30_), 4 plasmids (S_0_, S_10_, S_20_ and S_30_), or 7 plasmids (S_0_, S_5_, S_10_, S_15_, S_20_, S_25_ and S_30_), by the same proportion and performed the MS-MCA.

### Quantification of the degree of methylation in various DNA samples

DNA from relative pure cell samples, *e*.*g*. cell lines or our control plasmids, generally methylated in the close or same pattern. Thus, only single peaks appear in their corresponding curves and the average methylation degree can be measured. Using the series of control plasmids which imitating the bisulfite-converted DNA of different methylation degrees (Fig 1A and 1B), a standard curve was plotted and linearly fitted to establish the relation between the melting temperatures (Tm) of PCR products and the corresponding DNA template methylation degrees (S2 Fig). Then the methylation extent of an unknown DNA sample can be measured.

As for miscellaneous DNA samples, *e*.*g*. dissected tumor tissues or plasma samples, their corresponding MS-MCA results turned out to be multiple featured peaks. The unmethylation-featured peak which overlapped with S_U_ was ignored, and the remaining ones were measured accordingly as mentioned above.

### A semi-quantitative analysis for miscellaneous DNA samples

DNA from miscellaneous cell samples, *e*.*g*. dissected tumor tissues or plasma samples, generally composed of a collection of DNA methylated in various degrees, which makes the precise quantification of methylated DNA in a DNA pool quite difficult. To resolve this problem, we proposed a simplified model, *i*.*e*., regarding any miscellaneous DNA sample as composed of only two types of DNA, the non-methylated and fully-methylated ones, which were pooled together in a certain ratio. Then, the semi-quantification of methylated DNA proportion relative to the total DNA can be performed by using the following method.

There are two forms of melt curves: the original one represents the normalized fluorescence signals’ change following the elevation of temperature (Fig 1A), and the other is a derivative form of the original one, reflecting the change rate of the signals with the temperature (Fig 1B). The original form was used for semi-quantification. As illustrated above, the temperature interval between 75.5°C to 79.5°C was regarded as unmethylated, and the non-methylation-DNA-corresponding PCR products (N-products) unraveled during this stage, causing a decline in fluorescence signals, while the methylation-DNA-corresponding PCR products (M-products) remained double-stranded with fluorescence. Since the fluorescence intensity is in proportion to the population of the PCR products, thus, when we do integral for this reduced fluorescence intensity, the relative quantity of N-products can be measured. Similarly, the relative quantity of M-products can be measured by integral from temperature 79.5°C to 84.5°C.

Then, a series of plasmid standards, each of which was a mix of S_M_ and S_U_ plasmids in the different concentration ratios (1:1000, 1:100, 1:10, 1:4, 2:3, 1:1, 3:2, 4:1, 10:1, 100:1 or 1000:1, plus pure S_M_ or S_U_, respectively), were set to establish the correlation between initial methlylated DNA input and the final M-products obtained (S3 Fig). This standard curve can be used to roughly calculate the percentage of methylated DNA in the original DNA materials.

### ROC Curve in determining the methylation cut-off value

**Fig 2:**
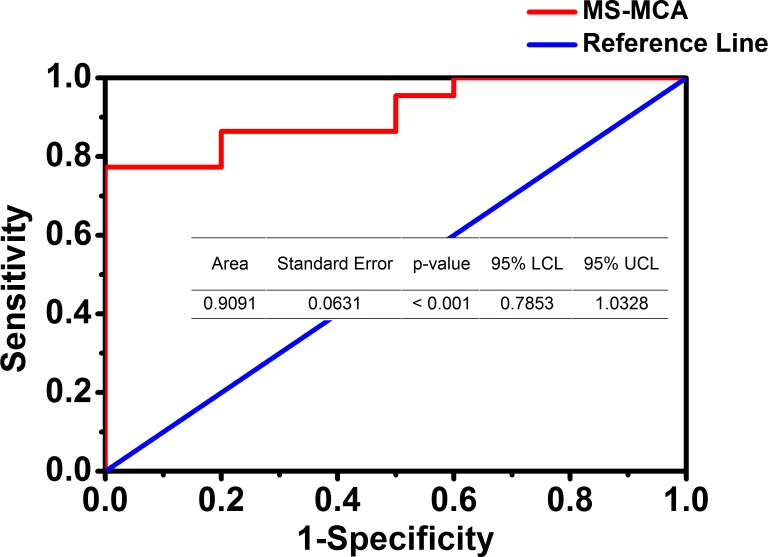
The ROC curve used to evaluate the MS-MCA method and set the methylation cut-off value.

Receiver-operating characteristic (ROC) plots have been widely used in evaluating clinical medicine effects and the diagnostic accuracy [17]. Thus, we collected a total of 32 clinical samples and performed both MS-MCA and bisulfite sequencing, as illustrated above. Then, we used the semi-quantification data of MS-MCA as test variables and bisulfite sequencing data as a gold-standard to determine the unmethylated or methylated DNAs, and plotted a ROC curve accordingly. The ROC curve and the corresponding parameters are given in Fig 2. After the curve was plotted, the Youden index was calculated from the dozens of points along with the curves by using the equation: Youden = Senstivity + Specificity -1. Then, the methylation cutoff value was set at 13.10% (of methylated DNA in a total DNA pool) where the maximum Youden value was found. In this case, the samples with starting methylated DNA content higher than the threshold would be regarded as methylated.

## Results

### Assessment of *RPRM* gene’s methylation in various types of samples

**Fig 3:**
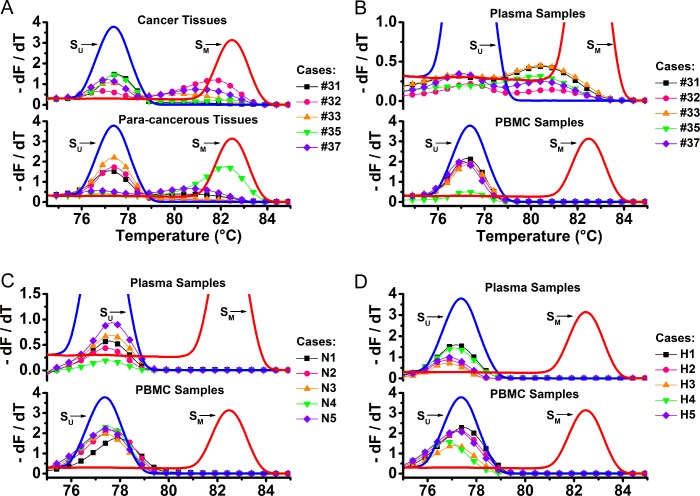
MS-MCA results of different types of samples from gastric cancer patients, non-tumor patients and healthy individuals. (A) MS-MCA results of gastric cancer tumor (upper panel) or the corresponding para-cancerous tissue samples (lower panel). (B) MS-MCA results of gastric cancer patients’ plasma (upper panel) or the PBMC samples (lower panel). (C) MS-MCA results of non-tumor patients’ plasma (upper panel) or the PBMC samples (lower panel). (D) MS-MCA results of healthy individuals’ plasma (upper panel) or the PBMC samples (lower panel). As controls, S_U_ and S_M_ are marked with bold lines, respectively.

Four types of specimens including the gastric cancer tumor tissues, para-cancerous tissues, plasma and PBMC samples were assessed. Five cases of gastric cancer patients’ specimens were selected as the representatives for displaying the MS-MCA results (Fig 3).

Most of the tumor samples resulted in two distinguishable peaks. One fell between the control plasmids’ peaks of S_U_ and S_M_, indicating the methylation signal, while the other overlapped with that of S_U_, indicating the non-methylation signal. However in some cases, none methylated peaks were found (Fig 3A, the upper panel, case #31 and #35). As for the corresponding para-cancerous tissues samples, quite a few also showed a positive methylation outcome (Fig 3A, the lower panel, case #35 and #37).

As for the gastric cancer patients’ plasma samples, nearly all of them were found with methylation (Fig 3B, the upper panel). However, most of their corresponding PBMC DNAs from the same whole blood samples were free from methylation modification (Fig 3B, the lower panel). In contrast, the methylation occurrence appeared less common in non-tumor patients’ or healthy adults’ plasma or PBMC samples (Fig 3C and 3D).

Using the semi-quantitative analysis illustrated in the Materials and Methods, each sample was assessed and classified into the unmethylated or methylated groups. And the statistic results were given in Table 1.

**Table 1:** Methylation Occurrence in Various Types of Samples.

Sample Types	Gastric Cancer			Non-tumor Plasma^a^	Healthy Plasma	All PBMC
	Ca. Tissues	Para. Tissues	Plasma			
**Samples**	42	28	35	29	28	41
**Methylated**	30	15	33	8	2	1^b^
**Met%**	70.0%	53.6%	94.3%	27.6%	7.1%	2.4%

^a^ Non-tumor plasma samples were collected from patients who were free from malignant tumor invasion, but diagnosed with gallstones, cholecystitis, and intestinal obstruction jaundice, *etc*.

^b^ The only one methylated case of PBMC samples came from a gastric cancer patient.

### Assessment of methylation heterogeneity in various types of samples

**Fig 4:**
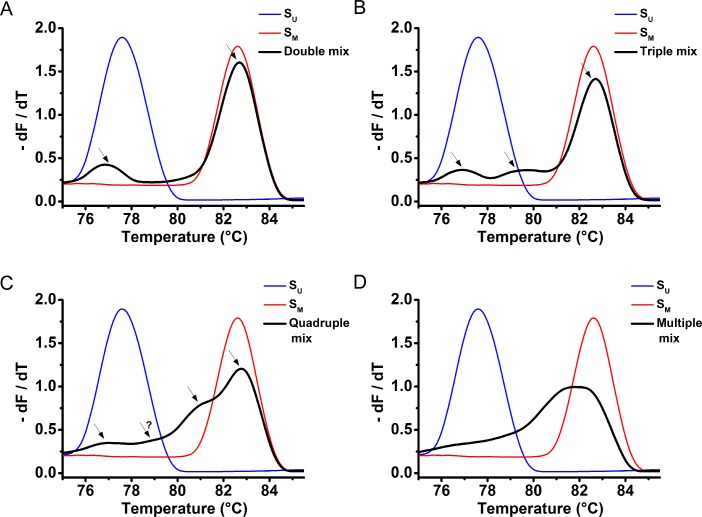
MS-MCA results of heterogeneous DNA pooled with different control plasmids. (A) The MS-MCA result of heterogeneous DNA mixed with S_0_ (S_U_) and S_30_ (S_M_) in the same proportion. (B) The MS-MCA result of heterogeneous DNA mixed with S_0_ (S_U_), S_15_ and S_30_ (S_M_) in the same proportion. (C) The MS-MCA result of heterogeneous DNA mixed with S_0_ (S_U_), S_10_, S_20_ and S_30_ (S_M_) in the same proportion. (D) The MS-MCA result of heterogeneous DNA mixed with S_0_ (S_U_), S_5_, S_10_, S_15_, S_20_, S_25_, and S_30_ (S_M_) in the same proportion. Visible peaks in these individual figures are marked with arrows, and as controls, curves of S_U_ and S_M_ are also indicated along.

To see if MS-MCA can be used in determining the DNA methylation heterogeneity of a sample, we pooled control plasmids in several different ways and generated the corresponding MS-MCA curves. As shown in Fig 4A and 4B), up to 3 peaks can be spotted in the curves; as for more intricate cases, the peaks overlap with each other to form a continuous broad peak (Fig 4C and 4D). This suggests a possibility in judging the heterogeneous and complex nature of a sample.

### The methylation degree in various methylated samples

**Fig 5:**
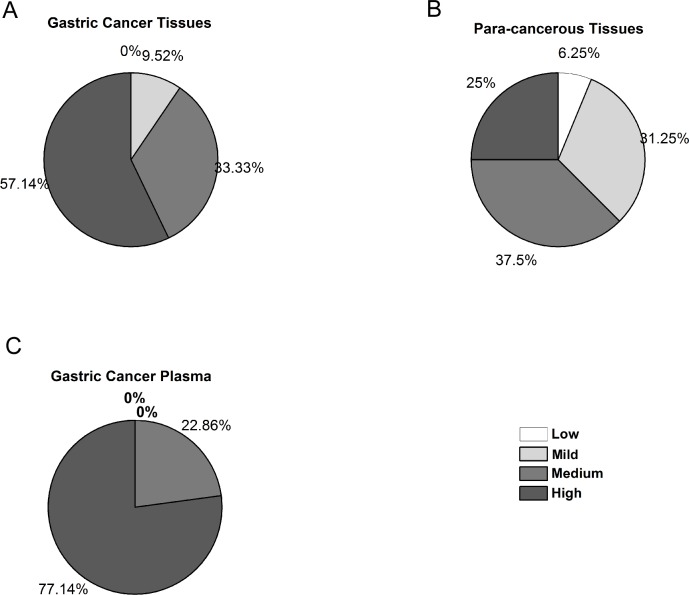
Distribution of the methylated samples according to the methylation degree. The methylation degree was divided into four groups, i.e. low (0–25%), mild (25–50%), medium (50–75%) and high (75–100%). Each methylated sample was calculated for a methylation degree and classified accordingly. (A) Distribution of gastric cancer tissues with the different methylation degrees. (B) Distribution of para-cancerous tissues with the different methylation degrees. (C) Distribution of gastric cancer patients’ plasma samples with the different methylation degrees.

The methylation degree of various methylated samples was quantified by using MS-MCA. We intentionally divided the different methylation degree into four classes: low (0–25%), mild (25–50%), medium (50–75%) and high (75–100%) methylation degrees. And then we picked methylated samples including the gastric cancer tissues, para-cancerous tissues and gastric cancer patients’ plasma samples, for classification. As shown in Fig 5A and 5B, for the gastric cancer tissue or the para-cancerous tissue samples, the methylation degrees varied greatly from unmethylated to almost fully-methylated degrees among different cases, although the gastric cancer tissue samples appeared to be methylated to a higher degree as compared with the corresponding para-cancerous tissues. As for those methylated plasma samples, however, they displayed a distinct methylation pattern which inclined to be consistently hypermethylated to a higher degree (Fig 5C).

### *RPRM* promoter methylation profile in gastric cancer cell lines

The bisulfite sequencing results of 62 cases samples (22 paired cases of gastric cancer tumor and the corresponding para-cancerous tissue samples, plus 18 cases of gastric cancer patients’ plasma samples) did not show any particular preferably methylated locus or session within the target sequence studied. This may be caused by the heterogeneity of cancerous samples.

**Fig 6:**
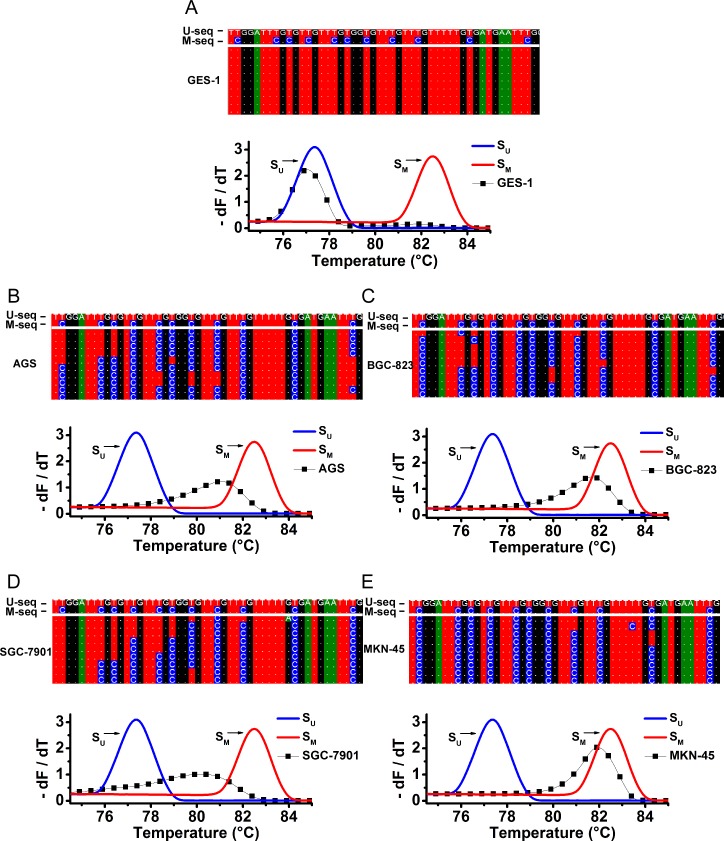
Bisulfite sequencing and MS-MCA results of different gastric (cancer) cell lines. Eight to ten clones obtained from each bisulfite-converted cell line DNA were sequenced and aligned with U-seq and M-seq. Only a part of the bisulfite sequencing alignment results was shown in these figures and methylated cytosines are highlighted in blue color. (A) Partial bisulfite sequencing results (the upper panel) and MS-MCA results (the lower panel) of GES-1 gastric epithelium cell line. (B) Partial bisulfite sequencing results (the upper panel) and MS-MCA results (the lower panel) of AGS gastric cancer cell line. (C) Partial bisulfite sequencing results (the upper panel) and MS-MCA results (the lower panel) of BGC-823 gastric cancer cell line. (D) Partial bisulfite sequencing results (the upper panel) and MS-MCA results (the lower panel) of SGC-7901 gastric cancer cell line. (E) Partial bisulfite sequencing results (the upper panel) and MS-MCA results (the lower panel) of MKN-45 gastric cancer cell line.

Therefore, we bisulfite sequenced the DNA from the gastric (cancer) cell lines including GES-1, AGS, BGC-823, SGC-7901 and MKN-45 to determine the precise methylation status of each cytosines. None methylated cytosines (0.0%, 0 in total 30 cytosines) were found in the bisulfite sequencing results of all clones from the GES-1 cell line. However, different methylation patterns were found for the cancer cell lines including AGS, BGC-823, SGC-7901 and MKN-45, with methylated cytosines of 83.3% (25/30), 91.3% (27.4/30), 43.7% (13.1/30) and 95.7% (28.7/30). These results were highly consistent with that quantified by using the MS-MCA method illustrated above (Fig 6 and Table 2).

**Table 2:** Different cell lines methylation degree determined by bisulfite sequencing (BS) or MS-MCA.

Gastric (Cancer) Cell Lines		GES-1^a^	AGS	BGC-823	SGC-7901	MKN-45
**Methylation Degree%**	**BS**	0.0%	83.3%	91.3%	43.7%	95.7%
	**MS-MCA**	0.0%	78.0%	87.6%	54.1%	93.4%

^a^ GES-1 is a non-malignant gastric epithelium cell line.

Intriguingly, the methylation patterns within the clones of a same cell line also proved to be differentiated. Some methylated to different degrees, while others methylated to the same degree but at a different location. These findings were also substantiated by the MS-MCA plots, where the methylation-corresponding peak appeared as the broader shapes compared with the controls, indicating the complex composition of the PCR products (Fig 6).

Therefore, for heterogeneous tissue samples, we believe that MS-MCA offers a more simplified methodology to provide the semi-quantitative information regarding the overall DNA methylation status of a gene in comparison with bisulfite sequencing.

## Discussion

### MS-MCA as an advantageous method in detecting DNA methylation in miscellaneous samples

#### Comparison with bisulfite sequencing technique

We established the MS-MCA technique to analyze DNA methylation in miscellaneous samples such as tumor tissues or plasma samples, and first used this method to relatively quantify methylated DNA contents. And we also used bisulfite sequencing to pinpoint the methylated cytosines within the target sequence. These two methods have their own advantages, where bisulfite sequencing picks sufficient amplicons to point out each cytosine’s methylation, while MS-MCA neglects the single cytosine’s methylation details but analyzes the bisulfite-amplified PCR products as a whole. Bisulfite sequencing has been reckoned as a gold standard for determining DNA methylation. In the study, it was also used to evaluate the accuracy of MS-MCA (Fig 2). However, when dealing with miscellaneous samples, for example, dissected tumor samples, which were composed of heterogeneously originated cells such as malignant tumor cells, normal epithelium cells or tumor infiltrating lymphocytes [18], *etc*., bisulfite sequencing is no longer effective due to a high complexity of PCR products and requires a substantial sample quantity to obtain a reliable result.

Additionally, MS-MCA also enables a fast track of the methylation degree variation when the DNA are subjected to demethylating agents’ treatment or undergoing intracellular methylation modification. For example, the de-methylation process was clearly monitored when AGS cells were treated with demethylating drug 5-aza-2'-deoxycytidine (S4 Fig). Thus, in this context, we believe that MS-MCA is more feasible to analyze a single gene or several selected genes for mixed samples.

#### Assessment of the methylation degree of a methylated sample

Although it is known that tumors incline to be hypermethylated in tumor suppressor genes, it remains unclear why each individual case was methylated in various degrees (Fig 3A), as was a similar circumstance found in each of the gastric cancer cell lines which were originally derived from different patients (Fig 6B, 6C, 6D and 6E). If the different methylation degree is associated to a certain pathological trait or specific gene mutations, this feature might be applied in helping tumor typing, but needs to be further verified.

#### Assessment of the homogeneity or heterogeneity a sample

According to the differential melt curves with the single peak, the double peaks or even the multiple peaks, it was able to tell the complex composition of the samples. For example, in most of the dissected tissue samples, the double peaks were frequently found on the MS-MCA curves, with one unmethylated peak corresponding to normal non-malignant cells and the other for cancer cells, as we speculated (Fig 3). For another instance, as shown in Fig 6D and 6E, the two cancer cell lines SGC-7901 and MKN-45 displayed the different peak shapes, a dispersed broad peak and a concentrated sharp shape, respectively. This suggests the differentiated methylation patterns between these two cell lines, where SGC-7901 cell population inclines to methylate differently among each other, while MKN-45 prefers a similar methylation pattern. This also suggests that DNA methylation is a dynamic process and its pattern can be changed during the tumor development.

#### Semi-quantification of methylated DNA content in miscellaneous samples

Here, we used 20 cases of PBMC samples and control plasmid S_M_ to determine the non-methylation or fully-methylated intervals, respectively. Several factors other than cytosine methylation, such as single nucleotide polymorphisms (SNPs) or Indels of the target sequence may also cause a shift of the MS-MCA curve. Thus, using the PBMC samples to set the non-methylation interval lowers the probability to misjudge an unmethylated DNA sample and better resembles the intrinsic circumstance. However, when setting the fully-methylated interval, S_M_ control plasmid was used since from our current existing experimental data, zero case was found to be completely methylated.

It is also interesting to note that there is a bias of amplification between S_U_ and S_M_, where the latter one is more preferable for amplification when they are mixed in an equal amount (S3A Fig or Fig 4), even the primers used are the same. This suggests another advantage of this method since it was able to pick up methylation signals even when the methylated DNA’s content in the initial DNA input was as low as 0.1% (see the zoomed in region in S3A Fig).

### *RPRM* promoter methylation is highly associated with gastric cancer

Methylation is an epigenetic modification highly associated with carcinogenesis. The aberrant *RPRM* gene methylation has been associated with dysfunction of the cell cycle and carcinogenesis [5, 19]. Our data revealed that in healthy population or normal functional cell types (such as PBMCs), the *RPRM* gene was less frequently methylated. However in gastric cancer patients, *RPRM* was frequently methylated in both tumor tissues and the corresponding para-cancerous tissues.

Interestingly, *RPRM* methylation was more frequently found (33 in a total 35 cases, Table 1) in the gastric cancer patients’ plasma, even when the tumor tissues from the same individual were not detected with methylation. A possible cause of this might be due to sampling inaccuracy, since we only sliced a small piece of tissues for DNA extraction, and might miss the most malignant parts. In this context, the methylated DNA in the plasma samples where circulating DNA from the whole body are enriched are hard to be missed.

Our studies support a possible application by using *RPRM* gene’s methylation in human plasma or serum samples to help diagnose gastric cancer or for prognosis, as suggested by Bernal *et al* [20]. According to their study, the methylation occurrence of *RPRM* gene in 32 retrospectively collected gastric cancer tissues was about 70%, similar to what we have observed in our study. In their study by using 43 prospectively collected gastric cancer cases, the *RPRM* gene methylation occurrence in tumor tissues and plasma were 97.7% and 95.3%, respectively, which are higher than our results. The discrepancy regarding this may be due to the difference of the patient groups or individuals used in the studies since our specimens were taken in China. Another reason may be due to the difference of the methodologies. Bernal *et al* used methylaiton-specific PCR (MSP) technique for detection of the *RPRM* gene methylation. Because the methylation-specific primers used in MSP only recognize very limited numbers of methylated cytosines (generally 2 to 5), it may overlook the possible methylation occurred beyond this region (i.e. false-negative results). In addition, MSP can also result in false-positive result. We previously found that the methylation-specific primers even with 4 mismatches to a template were able to amplify bisulfite-converted unmethylated DNA in late stage of PCR cycles. In contrast, the primers used in our MS-MCA analyses are independent of DNA methylation status and the MS-MCA analyzed 30 cytosine methylation sites as a whole. Therefore, our MS-MCA results maybe more precisely reflect the methylation occurrence of *RPRM* gene.

### The adjacent para-cancerous tissues have an indication for the pathological change

Tumor and the adjacent para-cancerous tissues were able to be distinguished according to their pathology. However, in the study, their boundary was indecisive in the perspective of DNA methylation, since the methylation was also frequently found in those para-cancerous specimens (Fig 3A, lower panel). It is possible that during the cancer development, the adjacent cells are also affected and respond to the malignant change. Alternatively, the epigenetic change such as DNA methylation takes place at an early stage, previous to a display of pathological features which can be observed by clinical imaging or immunohistochemistry. This suggests a possible application in using *RPRM* gene’s methylation for early diagnosis of gastric cancer.

### Heterogeneous nature of gastric cancer tumor tissues

In the study, many of the methylation-specific peaks in those MS-MCA results appeared as multiple individual peaks or a broad and wide peak formed by the conjunction of multiple peaks, which suggest that the heterogeneity of *RPRM* gene methylation was present in different types of samples including tissues and plasma. In plasma, this heterogeneity is conceivable because DNA might be released to the serum from all kinds of body cells. For tumor tissues, there are two possible explanations for this heterogeneity, one is that the tumor tissues are mixed with malignant tumor cells and normal epithelium cells, as well as tumor infiltrating lymphocytes; the other possibility is that even for malignant tumor population alone, the methylation patterns among the cells varied, as demonstrated by the different methylation patterns of SGC-7901 and MKN-45 cancer cell lines (Fig 6). For SGC-7901, the individual cells incline to behave differently in methylation patterns, suggesting that this cell type is undergoing differentiation accompanied with various degrees of DNA methylation, while MKN-45 cells favors a synchronous DNA methylation pattern. Whether this heterogeneity in *RPRM* gene methylation patterns represents for some pathological features needs to be further studied.

### Malignancy of tumor might be weighed by the methylation degree

For gastric cancer plasma, all of samples fell in the methylation degrees between 50 to 100%, with zero case found methylated in a lower degree. We are not sure if it is because only gravely methylated DNA could be released into the peripheral blood. As for the tissue samples, when comparing the gastric tumor tissues with the adjacent para-cancerous ones, it is interesting to find that malignant tumor tissues prone to being methylated in a more severe degree, while the adjacent para-cancerous tissues distribute towards a lighter extent. As we only assessed *RPRM* gene in this study, we don’t know whether other genes also undergo similar methylation patterns. But the results indicate that the malignancy degree of a tumor might be directly or indirectly associated with the DNA methylation degree.

## Supporting Information

S1 Fig MS-MCA results of non-methylation or fully-methylated samples used to find non-methylation or methylation region. (A) MS-MCA results of 20 cases of non-methylation PBMC samples. (B) MS-MCA results of 20 cases of fully-methylated S_M_ samples. Each of the thinner lines represents an independent sample case, and as controls, S_U_ and S_M_ are marked with bold lines, respectively.
(TIF)

S2 Fig The standard curve for the melt temperature (°C) of PCR products against the corresponding DNA template methylation degrees. (TIF)

S3 Fig The standard curve for the percentage of methylation-specific PCR products (M-products) against the percentage of initial methlylated DNA input. (A) and (B) MS-MCA results for S_M_ and S_U_ mixture with different ratios, and in (A), a region was zoomed in to visualize a weak methylation signal. (C) The standard curve for the percentage of methylation-specific PCR products (M-products) against the percentage of initial methlylated DNA input.
(TIF)

S4 Fig The *RPRM* gene methylation was reversed by the treatment of 5-Aza-2’-deoxycytidine in AGS cells. AGS cells were treated with 0 μM, 1 μM, 5 μM and 10 μM of 5-Aza-2’-deoxycytidine for 48 h. After the treatment, the DNA were extracted and the *RPRM* gene methylation was analyzed by MS-MCA as described in the main text.
(TIF)

S1 File Target sequence and bisulfite primers. (DOCX)
