# Supplementary figures and images for: Methylation-Sensitive Melt Curve Analysis of the Reprimo Gene Methylation in Gastric Cancer
Source: PLoS One. 2016 Dec 16;11(12):e0168635. doi: 10.1371/journal.pone.0168635 (PMC5161478; doi:10.1371/journal.pone.0168635)

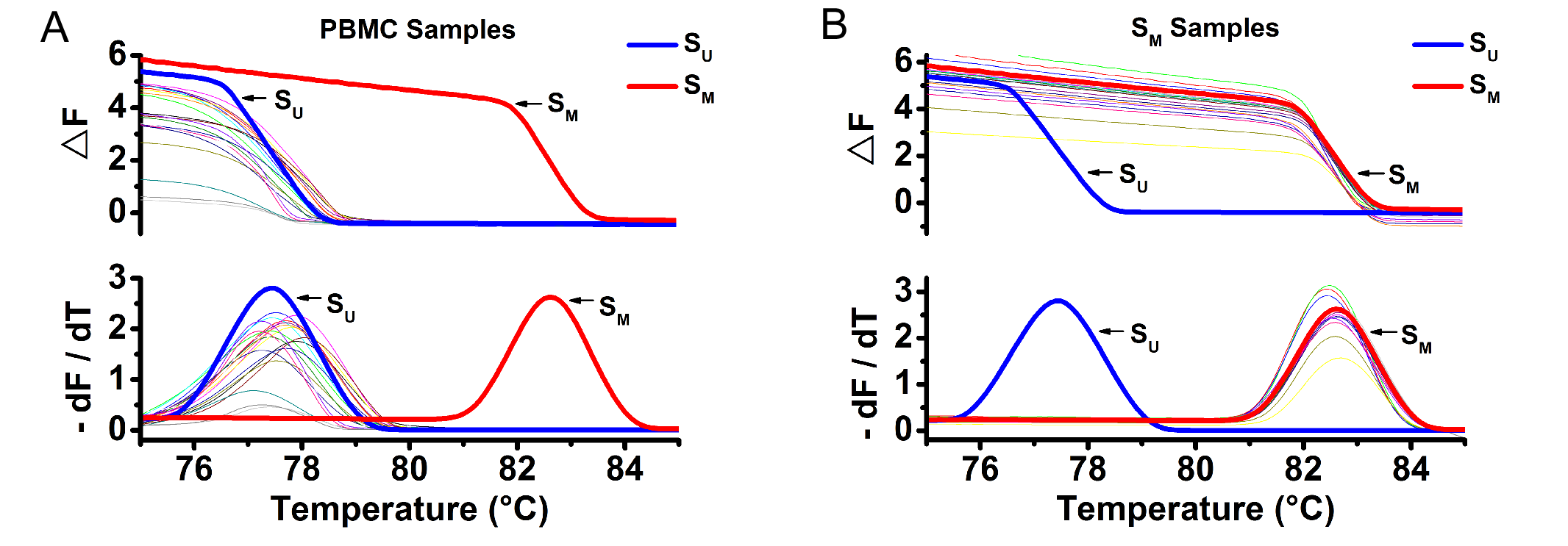

Supplement: S1 Fig — (A) MS-MCA results of 20 cases of non-methylation PBMC samples. (B) MS-MCA results of 20 cases of fully-methylated SM samples. Each of the thinner lines represents an independent sample case, and as controls, SU and SM are marked with bold lines, respectively. (TIF) [file pone.0168635.s001.tif]

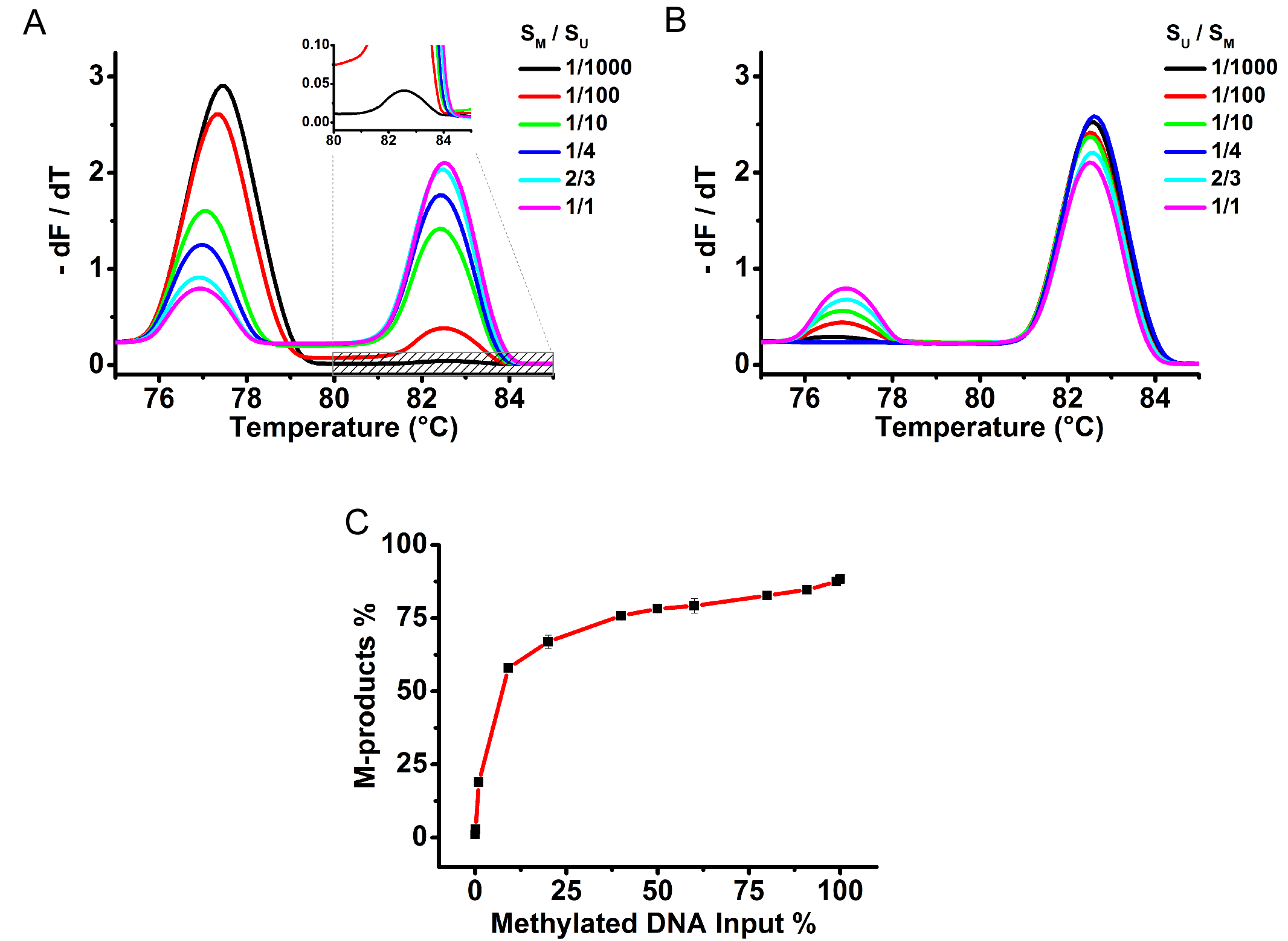

Supplement: S3 Fig — (A) and (B) MS-MCA results for SM and SU mixture with different ratios, and in (A), a region was zoomed in to visualize a weak methylation signal. (C) The standard curve for the percentage of methylation-specific PCR products (M-products) against the percentage of initial methlylated DNA input. (TIF) [file pone.0168635.s003.tif]
