# Supplementary material for: Methylation-Sensitive Melt Curve Analysis of the Reprimo Gene Methylation in Gastric Cancer
Source: PLoS One. 2016 Dec 16;11(12):e0168635. doi: 10.1371/journal.pone.0168635 (PMC5161478; doi:10.1371/journal.pone.0168635)
Supplement: S1 File — (DOCX) [file pone.0168635.s005.docx]

**Target sequence and bisulfite primers**

The targeted human *RPRM* gene sequence (Ref-seq) and the corresponding bisulfite-converted sequences from fully-methylated (M-seq) and unmethylated (U-seq) genomic DNA were indicated under below. Cytosines (C) were marked with blue color, and thymines (T) converted from cytosines were marked with red color. The bisulfite primers (RPRM-F: GTTTTAGAAGAGTTTAGTTGTTG; RPRM-R: CTACTATTAACCAAAAACAAAC) can be used to amplify bisulfite-treated genomic DNA sequences, referred to as M-seq and U-seq, respectively. The primer binding sites in M-seq and U-seq were underlined.

The targeted genomic sequence of human RPRM gene (Ref-seq):

**GCTTTAGAAGAGCCTAGCTGCTGCGCGCGTCGGAGAGGCTCCTGGGAAACTCCCACGGCCCAGGGACTTTCGAAAGCAGAGCGAGGAGCCCTCGCACGCGCTAGTCTGCGAGTGAGCGCTCAGCCCGGCACCTGTTCCTCCAGCGCCGCCGCCTTCCCACCCCTCGGACCCGCGCCGCTCGCGGCGCCCGCCCGTTCCTGCGATGAATCCGGCCCTAGGCAACCAGACGGACGTGGCGGGCCTGTTCCTGGCCAACAGCAG**

The bisulfite-converted sequences from fully-methylated genomic DNA (M-seq):

**GTTTTAGAAGAGTTTAGTTGTTGCGCGCGTCGGAGAGGTTTTTGGGAAATTTTTACGGTTTAGGGATTTTCGAAAGTAGAGCGAGGAGTTTTCGTACGCGTTAGTTTGCGAGTGAGCGTTTAGTTCGGTATTTGTTTTTTTAGCGTCGTCGTTTTTTTATTTTTCGGATTCGCGTCGTTCGCGGCGTTCGTTCGTTTTTGCGATGAATTCGGTTTTAGGTAATTAGACGGACGTGGCGGGTTTGTTTTTGGTTAATAGTAG**

The bisulfite-converted sequences from unmethylated genomic DNA (U-seq):

**GTTTTAGAAGAGTTTAGTTGTTGTGTGTGTTGGAGAGGTTTTTGGGAAATTTTTATGGTTTAGGGATTTTTGAAAGTAGAGTGAGGAGTTTTTGTATGTGTTAGTTTGTGAGTGAGTGTTTAGTTTGGTATTTGTTTTTTTAGTGTTGTTGTTTTTTTATTTTTTGGATTTGTGTTGTTTGTGGTGTTTGTTTGTTTTTGTGATGAATTTGGTTTTAGGTAATTAGATGGATGTGGTGGGTTTGTTTTTGGTTAATAGTAG**
